# Chronic pain–mental health comorbidity and excess prevalence of health risk behaviours: a cross-sectional study

**DOI:** 10.1017/S1463423624000070

**Published:** 2024-04-08

**Authors:** Sophie Lumley, Dahai Yu, Ross Wilkie, Kelvin P. Jordan, George Peat

**Affiliations:** 1 Primary Care Centre Versus Arthritis, School of Medicine, Keele University, Keele, Staffordshire, UK; 2 Centre for Applied Health & Social Care Research (CARe), Sheffield Hallam University, Sheffield, South Yorkshire, UK

**Keywords:** anxiety, chronic pain, comorbidity, depression, health risk

## Abstract

**Background:**

Chronic musculoskeletal pain and anxiety/depression are significant public health problems. We hypothesised that adults with both conditions constitute a group at especially high risk of future cardiovascular health outcomes.

**Aim:**

To determine whether having comorbid chronic musculoskeletal pain and anxiety/depression is associated with the excess prevalence of selected known cardiovascular health risk behaviours.

**Method:**

A cross-sectional survey of adults aged 35+ years randomly sampled from 26 GP practice registers in West Midlands, England. Respondents were classified into four groups based on self-reported presence/absence of chronic musculoskeletal pain (pain present on most days for six months) and anxiety or depression (Hospital Anxiety and Depression Score 11+). Standardised binomial models were used to estimate standardised prevalence ratios and prevalence differences between the four groups in self-reported obesity, tobacco smoking, physical inactivity, and unhealthy alcohol consumption after controlling for age, sex, ethnicity, deprivation, employment status and educational attainment. The excess prevalence of each risk factor in the group with chronic musculoskeletal pain–anxiety/depression comorbidity was estimated.

**Findings:**

Totally, 14 519 respondents were included, of whom 1329 (9%) reported comorbid chronic musculoskeletal pain–anxiety/depression, 3612 (25%) chronic musculoskeletal pain only, 964 (7%) anxiety or depression only, and 8614 (59%) neither. Those with comorbid chronic musculoskeletal pain–anxiety/depression had the highest crude prevalence of obesity (41%), smoking (16%) and physical inactivity (83%) but the lowest for unhealthy alcohol consumption (18%). After controlling for covariates, the standardised prevalence ratios and differences for the comorbid group compared with those with neither chronic musculoskeletal pain nor anxiety/depression were as follows: current smoking [1.86 (95% CI 1.58, 2.18); 6.8%], obesity [1.93 (1.76, 2.10); 18.9%], physical inactivity [1.21 (1.17, 1.24); 14.3%] and unhealthy alcohol consumption [0.81 (0.71, 0.92); –5.0%]. The standardised prevalences of smoking and obesity in the comorbid group exceeded those expected from simple additive interaction.

Chronic pain affects between one-third and one-half of the population of the UK (Fayaz *et al.*, 2016). Chronic musculoskeletal pain, defined as pain arising from muscles, bones, joints or surrounding soft tissues and persisting for more than three to six months (Treede *et al.*, 2015), makes up the majority of this burden (Breivik *et al.*, 2006; Goldberg and McGee, 2011; Fayaz *et al.*, 2016). One in six adults in the UK has a common mental disorder (comprising different types of depression and anxiety) (McManus *et al.*, 2016).

There is extensive evidence of the reciprocal relationship between chronic musculoskeletal pain and anxiety and/or depression, with the presence and severity of one, impacting the prevalence and trajectory of the other (Ruoff, 1996; Gambassi, 2009; Ang *et al.*, 2010; Fujii *et al.*, 2018; Mather *et al.*, 2019). Individually both chronic musculoskeletal pain and mental health disorders are associated with increased rates of cardiovascular disease (Oliveira *et al.*, 2020) and health risk behaviours which can negatively impact future morbidity and mortality, for example, increased smoking, low physical activity and unhealthy alcohol consumption (Scott and Happell, 2011; Van Hecke *et al.*, 2013). What is less clear is whether the combination of chronic musculoskeletal pain and anxiety/depression is associated with the excess prevalence of the key health risk behaviours associated with future cardiovascular disease and a range of other causes of morbidity and premature mortality.

Significant focus within primary care goes towards proactively identifying those at higher risk of future disease in order to target preventative advice and treatment, for example, in the UK, NHS Health Checks and opportunistic use of cardiovascular risk scores (National Health Service, 2020). Recent evidence from an evaluation of the NHS Health Check suggests that those with physical disability and those with mental illness do have equitable attendance for these checks (Patel *et al.*, 2020), but this analysis only specifically looked at attendance of those with ‘severe mental illness’, that is, those with schizophrenia, bipolar and other psychotic disorders, not depression and anxiety. Evidence suggests that those with mental illness, in general, are less likely to receive routine checks like blood pressure and cholesterol (Naylor *et al.*, 2016; Mental Health Foundation, 2021), and a lack of motivation, a symptom of mental illness, can serve as a barrier to effective behavioural change, even if health risk behaviours are discussed in primary care (Nordentoft *et al.*, 2013). In addition, there is some evidence to suggest that traditional lifestyle interventions may not be as effective in those with chronic pain and may need to be more intense or tailored to the individual (Van Hecke *et al.*, 2013). Given the high rates of health risk behaviour and potential under-recording of these behaviours in those with mental illness or chronic pain, it is possible that those with both morbidities may have an unrecognised additive or multiplicative prevalence, that is, higher rates of health risk behaviour then would be expected by adding or multiplying the prevalence in those with each isolated morbidity together. Understanding the prevalence in the comorbid group could serve as useful population health intelligence to aid general practice in proactively identifying and targeting health risk behaviours, when consulting with this group, or inviting patients for health checks. An understanding of the burden of health risk behaviours in this group could inform research to better understand how to tailor these interventions to ensure efficacy in this group.

## Research aims

1. To estimate the prevalence of obesity, smoking, unhealthy alcohol intake and inactivity, in those with comorbid chronic musculoskeletal pain and anxiety/depression and those with either/neither of these morbidities.
2. To explore whether any association between lifestyle factors and comorbid chronic musculoskeletal pain and anxiety/depression compared with those with each isolated morbidity is attenuated after adjustment for age, gender, ethnicity, deprivation, employment status and higher education.
3. To examine for interaction between chronic musculoskeletal pain and anxiety/depression in the association with risk behaviours (ie, to assess if any association between chronic musculoskeletal pain and anxiety/depression is greater than that expected by adding or multiplying the risks of the two individually).

To explore whether any association between chronic pain, anxiety/depression morbidity/comorbidity and health risk behaviours varied at different stages of adulthood.

## Methods

### Study design and setting

A cross-sectional survey of the general adult population aged 35 years and over registered with 26 general practices in West Midlands, England. Data were combined from three surveys (PRELIM Pilot, PRELIM Main and HILL) conducted between November 2017 and October 2019, which used similar methods and data collection instruments.

### Sampling, recruitment and data collection

This is a secondary analysis of data from three cross-sectional studies which set out to collate data on key patient-reported outcomes and psychosocial vital signs with a focus on common disabling musculoskeletal disorders. Adults over the age of 35 years and continuously registered at 26 participating West Midlands general practices for at least 10 years were selected using either random (PRELIM Main and HILL) or census (PRELIM Pilot) sampling. The derived sample was mailed a pen-and-paper questionnaire. Practices were selected to provide a range of deprivation and socio-economic status in these studies. Paper-and-pen questionnaires were mailed out to sampled patients using *Docmail©*. The study packs they received contained an invitation letter, the survey, a pre-paid envelope, a participant information leaflet and a contact number for a member of the research team to answer any further questions.

In PRELIM Pilot and Main, non-responders were contacted after two weeks with a duplicate study pack and the opportunity to complete the survey online. A further study pack was sent to non-responders at four weeks with an abridged ‘minimum data collection’ version of the survey. In HILL, non-responders were sent a duplicate pen-and-paper survey at three weeks. In both studies, non-responders at six weeks were presumed to have declined to participate.

### Study instruments

The surveys collected data on a wide range of health determinants and outcomes (a description of the full survey instrument can be found at Wilkie *et al*., 2020 ). The survey content was reviewed by members of Keele University’s Patient and Public Involvement and Engagement Research Users Group (RUG).

#### Chronic musculoskeletal pain and anxiety/depression

Chronic musculoskeletal pain was assessed using the item, ‘In the past 6 months, how often did you have pain’ (Dahlhamer, 2018) and defined as responding ‘most days’ or ‘every day’, consistent with recent recommendations (Kroenke *et al.*, 2019). We verified our assumption that this would be predominantly musculoskeletal in origin, by demonstrating that 92.4% of respondents classed as having chronic pain by this definition reported pain that was present for most/every day in the last six months in the back, neck, shoulder, hand/wrist, hip, knee or foot/ankle.

Anxiety and depression symptoms were assessed using the Hospital Anxiety and Depression Score (HADS). We defined the presence of anxiety/depression as a score of 11 or more on either subscale. A threshold of 11 has been used to ensure differentiation of ‘probable’ from ‘possible’ cases of depression or anxiety so that this definition focuses on those most likely to have clinically significant symptoms (Zigmond and Snaith, 1983; Hansson *et al.*, 2009).

#### Health risk behaviours

Current body mass index (BMI) was assessed by self-reported height and weight, and obesity was defined as BMI ≥30 kg/m^2^.

Self-reported smoking status was classed as ‘current’ versus ‘never’/’former’ (last smoked over 12 months ago).

Current physical activity was assessed using the General Practice Physical Activity Questionnaire (GPPAQ), a seven-item screening tool designed to assess physical activity in adults in primary care. Respondents were categorised as ‘inactive’, ‘moderately inactive’, ‘moderately active’ or ‘active’. For the current analysis, we dichotomised responses into ‘inactive’/‘moderately inactive’ versus ‘moderately active’/‘active’ (NHS, 2009).

Alcohol consumption was assessed by self-reported number of glasses of wine, pints of beer/cider or 25 ml measures of spirits per week, multiplied by the alcohol unit content. Alcohol intake of >14 units/week was classed as an unhealthy level of consumption (Department of Health and Social Care, 2016).

#### Covariates

Covariates included:

- Patient age (continuous variable)
- Gender
- Ethnicity (White, Black-Caribbean, Black-African, Black-Other, Indian, Pakistani, Bangladeshi, Chinese, and Other)
- Index of Multiple Deprivation (IMD) (Department for Communities and Local Government, 2016), an area-level measure which ranks all neighbourhoods (local super output area, mean population 1500) in England on relative deprivation based on multiple indicators from seven domains – income, employment, education, skills and training, health and disability, crime, barriers to housing and services, and living environment. For analysis, IMD rank associated with respondents’ residential address was categorised by quintile ranks
- Employment status, working-age participants (currently in paid employment or self-employed versus other)
- Educational attainment (school only versus full-time higher education or university).

### Data analysis

All participants who provided complete data on the presence of chronic musculoskeletal pain and anxiety or depression (96%) were included in the current analysis.

Survey respondents were classified into four mutually exclusive categories:

- Group 1 – Participants without chronic musculoskeletal pain, anxiety or depression
- Group 2 – Participants with anxiety or depression without chronic musculoskeletal pain
- Group 3 – Participants with chronic musculoskeletal pain without anxiety or depression
- Group 4 – Participants with chronic musculoskeletal pain and anxiety or depression comorbidity

The characteristics of each of the four groups were described.

To estimate whether the joint presence of chronic pain and anxiety/depression was associated with the excess prevalence (ie, positive interaction on additive and multiplicative scales) of health risk behaviours after controlling for covariates, we used standardised binomial models (Richardson *et al.*, 2015). The excess prevalence on additive and multiplicative scales was assessed after standardising for age, sex, ethnicity (White versus Black, Asian and other ethnicities), deprivation and educational attainment. Additive interaction was assessed using the relative excess risk due to interaction, that is, the absolute excess prevalence in the comorbid group, beyond what we would expect if there was no interaction, reported as a difference in prevalence where prevalence is expressed as a percentage of the population, and multiplicative interaction using the ratio of prevalence ratios, that is, how many times higher the prevalence was in the comorbid group compared with what we would expect if there was no interaction between chronic musculoskeletal pain and anxiety/depression. Exploratory analyses were then performed stratified by age categories (35–49, 50–64 and 65+ years), to explore whether the combination of chronic pain and anxiety/depression varied in association with health risk behaviours at different stages in adulthood. Confidence intervals for standardised estimation were calculated by bootstrapping with 10 000 resampling. All analyses were conducted using SPSS statistics v26 and Stata v14.2.

## Results

### Study participants

**Figure 1.**
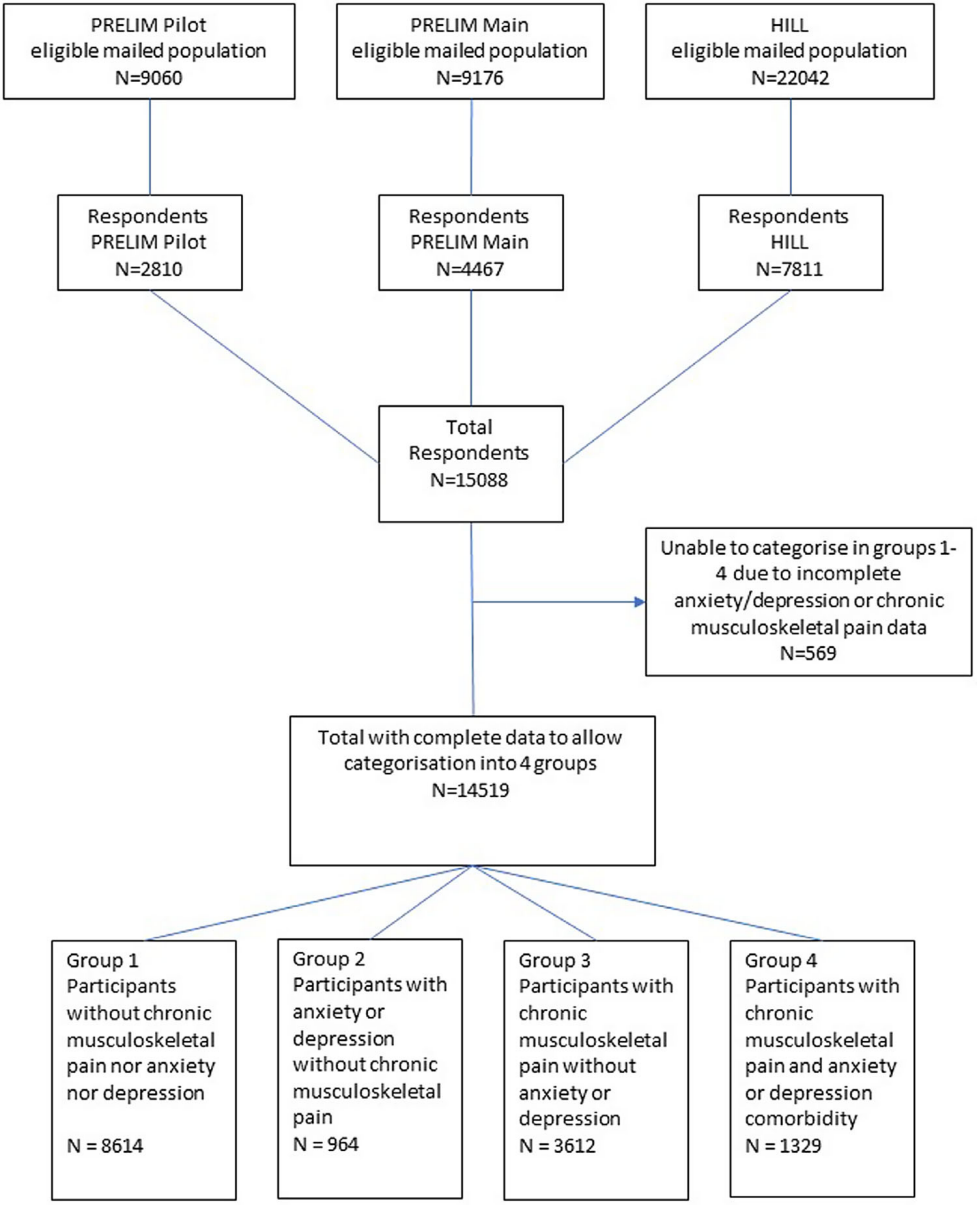
Participant flowchart

**Figure 2.**
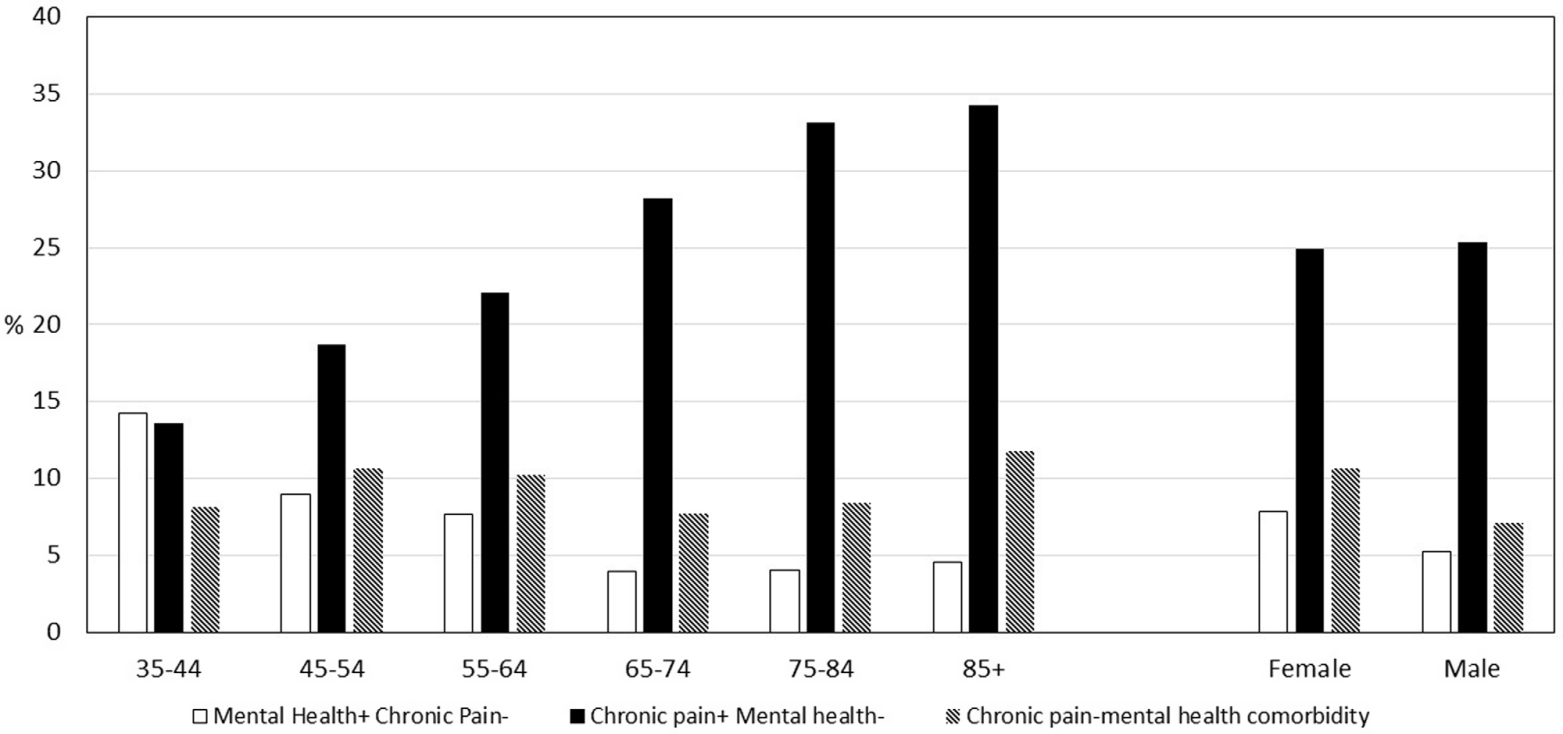
Crude prevalence of comorbid and non-comorbid chronic pain and mental health, by age and sex: West Midlands, 2017–2019

Totally, 14 519 participants (adjusted response rate 37.8%) were included in this analysis (Figure 1). The mean age was 64 (standard deviation (SD) 13) years: 56% were female; 4% were from Black, Asian or other ethnic minority groups; and 16% were living in one of the most deprived quintile areas in the UK. The crude prevalence of chronic musculoskeletal pain was 34% overall and increased with age. The crude prevalence of anxiety/depression was 16%. The crude prevalence of comorbid chronic pain and anxiety/depression was 9% (Figure 2). Of those with chronic pain, 91.7% had pain in >1 musculoskeletal site. The most common sites were back (78.3%), knee (70.4%) and shoulder (60.8%).

### Descriptive characteristics of adults with comorbid chronic musculoskeletal pain and anxiety/depression

Respondents in the comorbid group had the same mean age as those in the group with neither morbidity (63 years), though were slightly younger than those with isolated chronic musculoskeletal pain and older than those with isolated anxiety/depression. Those with comorbidity and isolated anxiety/depression had a higher percentage of female respondents (66%). There was a marked difference in deprivation between the groups, with double the proportion of the comorbid group in the most deprived quintile compared with those in the group with neither morbidity (26% versus 13%) (Table 1).

**Table 1.** Characteristics of population subgroups of adults aged over 35 years defined by chronic pain and anxiety/depression, West Midlands, 2017–2019

	Population subgroup							
	Absent		Absent		Present		Present	
Chronic musculoskeletal pain	Absent		Present		Absent		Present	
Anxiety and/or depression	*n*	%	*n*	%	*n*	%	*n*	%
Respondents (% of total)	8614	59	964	7	3612	25	1329	9
Age (years): mean (SD)	63(13)		58(13)		67(12)		63(13)	
Age category (years):								
35–44	762	9	169	18	162	4	97	7
45–54	1,626	19	236	25	491	14	280	21
55–64	2,092	24	268	28	770	21	355	27
65–74	2,516	29	166	17	1,180	33	323	24
75–84	1,323	15	98	10	805	22	204	15
85+	295	3	27	3	204	6	70	5
Female	4,556	54	628	66	2,007	56	857	66
Black, Asian and Minority Ethnicity	338	4	48	5	126	4	57	4
Area-level deprivation:								
(Most) 1	1,117	13	155	17	620	18	334	26
2	1,381	16	162	17	619	18	268	21
3	1,654	20	169	18	660	19	245	19
4	2,414	29	288	31	1,000	28	270	21
(Least) 5	1,835	22	158	17	638	18	177	14
Employment status [working-age participants (≤65years) currently in paid employment or self-employed]	3,623	77	507	76	1,026	69	384	51
Higher education (full-time education after school)								
Yes	2650	32	382	40	820	24	312	24
No	5564	68	580	60	2579	76	1009	76
Lifestyle characteristics								
Obesity (BMI >30 kg/m^2^)	1553	20	211	23	1002	32	492	41
Smoking (current smoker)	624	8	97	10	270	8	192	16
Unhealthy alcohol consumption (>14 units/week)	2161	27	206	22	735	22	238	18
Inactivity (GPPAQ – inactive or moderately inactive)	5558	68	662	69	2662	78	1108	83

BMI = body mass index; GPPAQ = General Practice Physical Activity Questionnaire.

### Health risk behaviours in adults with comorbid chronic musculoskeletal pain and anxiety/depression

Of the four groups, adults with comorbid chronic musculoskeletal pain and anxiety/depression had the highest crude prevalence of obesity (41%), current smoking (16%) and physical inactivity (83%). The crude prevalence of unhealthy alcohol consumption was highest in the group with neither chronic musculoskeletal pain nor anxiety/depression (Table 1).

After standardising to the age, sex, ethnicity, deprivation and educational attainment profile of the respondent population (Supplementary Table 1), those with comorbid chronic musculoskeletal pain and anxiety/depression were almost twice as likely to be obese (39% versus 20%) and to currently smoke (15% versus 8%) than adults with neither chronic musculoskeletal pain nor anxiety/depression [standardised prevalence ratios of 1.93 (95% CI 1.76, 2.10) and 1.86 (1.58, 2.18) respectively]. They were 1.21 (1.17, 1.24) times as likely to be inactive or moderately inactive; however, they were less likely [standardised prevalence ratio of 0.81 (95 CI 0.71, 0.92)] to consume an unhealthy number of alcohol units (Table 2; Supplementary Table 2).

**Table 2.** Standardised prevalence of health risk behaviours in adults with chronic musculoskeletal pain and anxiety/depression comorbidity compared with adults with neither chronic musculoskeletal pain nor anxiety/depression

	Standardised^a^ prevalence in adults with neither chronic pain nor anxiety/ depression (Group 1)	Standardised prevalence in adults with chronic pain and anxiety/ depression (Group 4)	Standardised prevalence difference	Standardised prevalence ratio	Additive interaction	Multiplicative interaction
Obese (BMI >30 kg/m^2^)	20.3%	39.2%	18.9 (15.7, 22.0)	1.93 (1.76, 2.10)	5.5 (0.3, 9.8)	1.14 (0.94, 1.34)
Current smoker	7.9%	14.7%	6.8 (4.6, 8.9)	1.86 (1.58, 2.18)	4.7 (1.2, 7.7)	1.46 (1.0, 1.91)
Unhealthy alcohol consumption (>14 units alcohol/week)	26.0%	20.9%	–5.1 (–7.7, 2.3)	0.81 (0.71, 0.92)	1.0 (–3.2, 5.3)	1.03 (0.83, 1.23)
Physically inactive (GPPAQ score inactive or moderately inactive)	69.0%	83.4%	14.4 (11.9, n16.7)	1.21 (1.17, 1.24)	3.8 (–1.0, 7.9)	1.04 (0.88, 1.21)

BMI = body mass index; GPPAQ = General Practice Physical Activity Questionnaire.

a Standardised for age, gender, ethnicity, deprivation and higher education.

Additive interaction = relative excess risk due to interaction, for example, interpreted as 5.5% higher prevalence of obesity in adults with comorbid chronic musculoskeletal pain and anxiety/depression than would be expected if there was no interaction between chronic musculoskeletal pain and anxiety/depression.

Multiplicative interaction = rate of prevalence ratios, for example, interpreted as 1.14 times higher prevalence of obesity in adults with comorbid chronic musculoskeletal pain and anxiety/depression than what would be expected if there was no interaction.

There was evidence on the additive scale of the excess prevalence of obesity and smoking and on a multiplicative scale of smoking due to interaction between chronic musculoskeletal pain and anxiety/depression. For example, the prevalence of obesity in adults with the comorbidity was 5.5% (95% CI 0.3, 9.8) higher than expected if there was no interaction between chronic musculoskeletal pain and anxiety/depression, and the relative risk of being a current smoker was 1.46 (95% CI 1.00, 1.91) times higher than would be expected by simply multiplying the risk in those with just anxiety/depression with the risk in those with just chronic musculoskeletal pain. No significant excess risk was seen for unhealthy alcohol consumption or physical activity (Table 2; Supplementary Table 2).

#### Stratification by age

In our exploratory analysis, the excess prevalence of obesity, smoking and physical inactivity due to interaction between chronic musculoskeletal pain and anxiety/depression appeared to be highest in younger age categories (Supplementary Table 3).

## Discussion

### Summary of findings

Our cross-sectional study of over 14 500 adults found that respondents with chronic musculoskeletal pain–anxiety/depression comorbidity have significantly higher prevalence of obesity, smoking and, to a lesser extent, physical inactivity than respondents with neither condition. These differences persisted after controlling for differences in age, sex, ethnicity and selected individual- and area-level socio-economic indicators. We found a positive interaction between chronic musculoskeletal pain and anxiety/depression, demonstrating the excess prevalence of obesity and smoking above what would be expected from simply adding the independent risks associated with chronic musculoskeletal pain and anxiety/depression alone. Contrary to expectations, we found no increased likelihood of unhealthy alcohol consumption in adults with comorbid chronic musculoskeletal pain and anxiety/depression.

### Study findings in the context of previous literature

This analysis presents new evidence from a large, regional and general population sample of the sociodemographic, pain and health risk characteristics of those with comorbid chronic musculoskeletal pain and anxiety/depression, compared with those without. In the absence of any information on non-responders, we were unable to model the associations for the entire target population. By comparison to survey non-response, missing data among responders on health risk behaviour, covariates, chronic pain and anxiety/depression were low (0%–11.3%), with the exception of missing data for alcohol consumption which was an outlier with 26%. The prevalence of chronic musculoskeletal pain (34%) in this study is comparable with the prevalence estimates in the literature. Fayaz *et al*. estimated the rates of chronic pain in UK adults to be 35%–51%, based on pooled data from seven studies, though they used a less strict definition of pain for >3 months (not >6 months), and included estimates of chronic widespread and other non-musculoskeletal pain (Fayaz *et al.*, 2016). The prevalence of depression and/or anxiety in this study (16%) is also comparable with the prevalence estimates in the UK; the Adult Psychiatric Morbidity Survey 2014 estimated one in six UK adults to have a common mental disorder (McManus *et al.*, 2016). Compared with 2011 Census estimates for the West Midlands, adults from Black, Asian and ethnic minorities were under-represented among survey respondents (4% versus 17%), and so too, but to a lesser extent, were men (41% versus 49%). It is also likely that older age groups are over-represented in this sample, with 50% of participants being over 65 years. However, given increased rates of chronic pain in older age groups, the findings of this study are highly relevant to this age group. Findings have been stratified by age to allow for some exploratory granular analysis of age-specific associations. The impact of lack of representativeness on prevalence estimates and interactions in this study is difficult to predict. However, over-representation of women and those >65 years of aged could have led to higher estimates of obesity and reduced estimates of smoking and alcohol (NHS Digital, 2020).

In self-reported surveys, there is a risk of information/reporting bias, given the subjective nature of data collection; however, there is a particular possibility of under-reporting when asking about risk behaviours, which are potentially stigmatising or clearly associated with negative health impacts (Livingston and Callinan, 2015; Norwood *et al.*, 2016). It is possible that there is under-reporting of alcohol consumption, smoking, activity levels and weight, though provided this is not particularly the case for one group within the analysis, the standardised prevalence ratios and differences would still be valid. The most recent government obesity statistics state that 28% of UK adults are obese (Baker, 2021), which is comparable to the 23% of participants in this study. Concerningly, the prevalence of obesity in the group with chronic musculoskeletal pain–anxiety/depression comorbidity was much higher at 41%. The prevalence of smoking in this study (9%) is comparable to the known UK prevalence of 14% (Office of National Statistics, 2019) from 2019 data. In the UK, 24% of adults are anticipated to regularly drink >14 units/week (PHE, 2016), which closely aligns with this study population where 23% of participants report drinking over recommended limits. There was no increased likelihood of unhealthy alcohol consumption in the comorbid group in our analysis. This may demonstrate that other unmeasured factors are more highly associated with drinking above recommended limits in this population or that we are seeing some effects of non-random under-reporting of weekly consumption. It is also possible that those with comorbidity are taking medications that may preclude them from unhealthy alcohol consumption. Government data from 2017/2018 estimate that 25% of people aged 16 years+ in England are physically inactive, doing <30 min of moderate-intensity activity a week (Department for Digital, Culture, Media and Sport, 2019). This prevalence is much lower than the 69% of participants in this study classed as inactive or moderately inactive. The most likely reason for this is differences in measurement. Our definition included the moderately inactive group of participants who do <1 h of exercise, so the definition is wider for ‘inactive’ then that is used in gov.uk statistics.

Despite a large number of respondents, numbers available for age-stratified analyses were limited and so must be treated as exploratory. Calculating 95CI for point estimates was time-consuming and computationally intensive and so we omitted this for the exploratory estimates. The suggestion that excess health risk behaviours may be greatest in working-age adults could have important implications for preventative action and should be investigated further in future adequately powered studies.

### Implications for research and practice

Our findings indicate that chronic musculoskeletal pain and anxiety/depression comorbidity is associated with a higher prevalence of some cardiovascular health risk behaviours, specifically obesity and smoking, than adults with either or neither. The use of standardised binomial modelling to demonstrate a positive additive/multiplicative interaction between chronic musculoskeletal pain and anxiety/depression helps to highlight this comorbid group as having excess risk, after standardising for potential confounders, and should be a target group for intervention. It is unclear whether the physical health needs of this group are being under-recognised in primary care or missed by routine opportunities to target cardiovascular risk, but evidence suggests that those with mental illness, in general, are less likely to receive routine checks like blood pressure and cholesterol (Naylor *et al.*, 2016; Mental Health Foundation, 2021). This study identified the need to ensure primary care assessment of cardiovascular risk and intervention in the anxious/depressed, particularly when they also have chronic musculoskeletal pain. There may be problems with those with comorbidity accessing, using and benefitting from recommended interventions for obesity reduction and smoking cessation, and more research exploring differential uptake in these services would be useful.

Over the last few years, despite increasing recognition of the importance of preventative medicine, in the context of cardiovascular risk, there has been widespread decommissioning of services directed at reducing risk. For example, between 2014/2015 and 2017/2018, local authority spending on tobacco control and stop smoking services in England fell by 30% (Cancer Research UK and Action on Smoking and Health, 2019). This is a problem when specialist, supervised stop smoking services have some of the best evidence from sustained smoking cessation (Bauld *et al.*, 2010). This may present a particular disadvantage for adults with comorbid chronic musculoskeletal pain and anxiety/depression, who have a higher likelihood of being a current smoker.

### Conclusion

The study highlights an interaction between comorbid chronic musculoskeletal pain and anxiety/depression leading to high and excess prevalence of some health risk behaviours associated with increased cardiovascular risk. This presents not only a public health problem but also a potential target group for opportunistic assessment of health risk in primary care and intervention to reduce future cardiovascular disease. Further research into recognition, recording and management of poor health risk behaviours among adults with chronic pain–mental health comorbidity, and increased understanding of access to, and efficacy of, interventions to reduce cardiovascular risk in this group, could help facilitate important decisions around how primary care and public health initiatives can be targeted in this high-risk group.

## Supporting information

Lumley et al. supplementary material Lumley et al. supplementary material

## Supplementary material

The supplementary material for this article can be found at https://doi.org/10.1017/S1463423624000070.
