# Supplementary material for: Chronic pain–mental health comorbidity and excess prevalence of health risk behaviours: a cross-sectional study
Source: Prim Health Care Res Dev. 2024 Apr 8;25:e15. doi: 10.1017/S1463423624000070 (PMC11022513; doi:10.1017/S1463423624000070)
Supplement: Lumley et al. supplementary material [file S1463423624000070sup001.docx]

**Supplementary Data**

**Supplementary Table 1. Characteristics of the four groups, before and after standardised weighting**

|  | **Population subgroup** | | | |
| --- | --- | --- | --- | --- |
| **Chronic pain** | **Absent** | **Absent** | **Present** | **Present** |
| **Anxiety and/or depression** | **Absent** | **Present** | **Absent** | **Present** |
| **Observed data** |  |  |  |  |
| Age (years): mean | 63 | 58 | 67 | 63 |
| Female | 53% | 66% | 56% | 66% |
| Higher education | 32% | 40% | 24% | 23% |
| Deprivation | 13% | 16% | 17% | 26% |
| Non-White ethnicity | 4% | 5% | 4% | 4% |
| **Weighted data** |  |  |  |  |
| Age (years): mean | 64 | 64 | 64 | 64 |
| Female | 56% | 58% | 56% | 55% |
| Higher education | 30% | 31% | 31% | 26% |
| Deprivation | 16% | 14% | 15% | 17% |
| Non-White ethnicity | 4% | 4% | 4% | 4% |

**Supplementary Table 2. Prevalence of selected health risk behaviours by chronic pain and anxiety/depression, expressed as standardised prevalence differences and prevalence ratios**

|  | **Population subgroup** | | | |
| --- | --- | --- | --- | --- |
| **Chronic pain** | **Absent** | **Absent** | **Present** | **Present** |
| **Anxiety and/or depression** | **Absent** | **Present** | **Absent** | **Present** |
| **N=** | 8614 | 964 | 3612 | 1329 |
|  | **Obesity** | | | |
| N case / controls | 1492/ 5826 | 200 / 653 | 967 / 2052 | 462 / 668 |
| Crude prevalence | 0.204 | 0.234 | 0.320 | 0.409 |
| Standardised prevalence (95%CI) | 0.203 (0.194,0.213) | 0.216 (0.187, 0.245) | 0.325 (0.308, 0.343) | 0.392 (0.362, 0.4) |
| Standardised prevalence difference (95%CI) | 0 | 0.012 (-0.019 to 0.043) | 0.121 (0.102 to 0.142) | 0.189 (0.157 to 0.22) |
| Standardised prevalence ratio (95%CI) | 1 | 1.06 (0.92, 1.22) | 1.60 (1.49, 1.71) | 1.93 (1.76, 2.1) |
| Measure of interaction on additive scale: absolute excess risk due to interaction (95%CI) = (0.189-0.121-0.012) = 0.055 (0.003 to 0.098) | | | | |
| Measure of interaction on multiplicative scale: ratio of standardised prevalence ratios (95%CI) = (1.93/ (1.6x1.06) = 1.14 (0.94 to 1.34) | | | | |
|  | **Current smoker** | | | |
| N case / controls | 592/7051 | 92/787 | 260/2901 | 184/1013 |
| Crude prevalence | 0.077 | 0.105 | 0.082 | 0.154 |
| Standardised prevalence (95%CI) | 0.079 (0.073,0.085) | 0.092 (0.072, 0.112) | 0.087 (0.076,0.097) | 0.147 (0.126,0.167) |
| Standardised prevalence difference (95%CI) | 0 | 0.013 (-0.008 to 0.034) | 0.008 (-0.005 to 0.02) | 0.068 (0.046 to 0.089) |
| Standardised prevalence ratio (95%CI) | 1 | 1.16 (0.93, 1.46) | 1.10 (0.95, 1.27) | 1.86 (1.58, 2.18) |
| Measure of interaction on additive scale: absolute excess risk due to interaction (95%CI) = 0.047 (0.012 to 0.077) | | | | |
| Measure of interaction on multiplicative scale: ratio of standardised prevalence ratios (95%CI) = 1.46 (1.00 to 1.91) | | | | |
|  | **Hazardous/harmful alcohol consumption** | | | |
| N case / controls | 2084/5660 | 198/697 | 714/2503 | 227/998 |
| Crude risk | 0.269 | 0.221 | 0.221 | 0.185 |
| Standardised risk (95%CI) | 0.260 (0.249,0.269) | 0.225 (0.195,0.255) | 0.234 (0.219, 0.250) | 0.209 (0.184,0.234) |
| Standardised risk difference (95%CI) | 0 | -0.034 (-0.066 to -0.003) | -0.025 (-0.043 to -0.007) | -0.05 (-0.077 to -0.023) |
| Standardised risk ratio (95%CI) | 1 | 0.867 (0.755,0.996) | 0.904 (0.838, 0.975) | 0.808 (0.713,0.916) |
| Measure of interaction on additive scale: absolute excess risk due to interaction (95%CI) = 0.01 (-0.032 to 0.053) | | | | |
| Measure of interaction on multiplicative scale: ratio of standardised risk ratios (95%CI) = 1.03 (0.83 to 1.23) | | | | |
|  | **Physical inactivity** | | | |
| N case / controls | 5298/2539 | 624/285 | 2545/716 | 1049/203 |
| Crude risk | 0.676 | 0.686 | 0.780 | 0.838 |
| Standardised risk (95%CI) | 0.690 (0.680, 0.701) | 0.755 (0.727, 0.783) | 0.732 (0.715, 0.749) | 0.834 (0.812, 0.856) |
| Standardised risk difference (95%CI) | 0 | 0.064 (0.035 to 0.093) | 0.042 (0.022 to 0.061) | 0.143 (0.119 to 0.167) |
| Standardised risk ratio (95%CI) | 1 | 1.093 (1.05, 1.137) | 1.06 (1.031, 1.089) | 1.208 (1.172, 1.244) |
| Measure of interaction on additive scale: absolute excess prevalence due to interaction (95%CI) = 0.038 (-0.006 to 0.079) | | | | |
| Measure of interaction on multiplicative scale: ratio of standardised risk ratios (95%CI) = 1.04 (0.88 to 1.21) | | | | |
| Standardised for age, sex, higher education, deprivation, ethnicity | | | | |

**Supplementary Table 3. Additive and multiplicative interaction for health risk behaviours, stratified for age group**

|  | Age group (years) | | |  | Age group (years) | | |
| --- | --- | --- | --- | --- | --- | --- | --- |
|  | 35-49 | 50-64 | 65+ |  | 35-49 | 50-64 | 65+ |
|  | Additive interaction† | | |  | Multiplicative interaction‡ | | |
| **Health Risk** |  |  |  |  |  |  |  |
| Obese (BMI >30) | 0.06 | 0.12 | 0.00 |  | 1.10 | 1.36 | 0.94 |
| Current smoker | 0.09 | 0.08 | 0.01 |  | 1.56 | 1.76 | 1.12 |
| Harmful/hazardous alcohol consumption (> 14 units alcohol/week) | 0.06 | 0.00 | -0.01 |  | 1.29 | 1.00 | 0.95 |
| Physically inactive  (GPPAQ‡ score inactive or moderately inactive) | 0.09 | 0.05 | 0.00 |  | 1.14 | 1.07 | 1.00 |
| BMI Body Mass Index; GPPAQ General Practice Physical Activity Questionnaire  †Additive interaction = relative excess risk due to interaction [RERI), e.g. interpreted as 6% higher prevalence of obesity in 35-49year olds with comorbid CMP and anxiety/depression than would be expected if there was no interaction between CMP and anxiety/depression  ‡Multiplicative interaction = ratio of prevalence ratios (ROR), e.g. interpreted as 1.76 times higher risk of being a current smoker in 50-64 year olds with comorbid CMP and anxiety/depression then would be expected if there was no interaction between CMP and anxiety/depression | | | | | | | |
